
JOURNAL INFORMATION
==============================
NLM Title Abbreviation: ScientificWorldJournal
Journal ID: ScientificWorldJournal
NLM Journal ID: 101131163
Journal ID: TSWJ

The Scientific World Journal

ISSN: 2356-6140
EISSN: 1537-744X
Publisher: Wiley

ARTICLE INFORMATION
==============================
PMCID: PMC6084267
PMID: 30147614
DOI: 10.1100/tsw.2001.239
Article ID: 948130
Article version: 1
Subjects: Short Report

Polyomavirus Large T Antigen Interact with the DISC and Protect against Fas Induced Apoptosis

Rodier F. 1
Bertrand R. 1 2
Mes-Masson A-M. 1 2 *
1 Centre de recherche CHUM, Hôpital Notre Dame and Institut du cancer de Montréal, 1560 rue Sherbrooke est, Montréal, Québec, Canada H2L 4M1,
2 Département de médecine, Université de Montréal, Montréal, Québec, Canada
*A-M. Mes-Masson: anne-marie.mes-masson@umontreal.ca
Publication date: 2001
Electronic publication date: 2001 Nov 22
Volume: 1
First page: 136
Last page: 136
Copyright: Copyright © 2001 F. Rodier et al.
Copyright year: 2001
License: This is an open access article distributed under the Creative Commons Attribution License, which permits unrestricted use, distribution, and reproduction in any medium, provided the original work is properly cited.
License URL: https://creativecommons.org/licenses/by/3.0/

Funding: Medical Research Council of Canada

==============================
Miami *Nature Biotechnology* Short Reports

*TheScientificWorld* (2001) 1 (S3), 136SR

ISSN 1532-2246; DOI 10.1100/tsw.2001.239

### POLYOMAVIRUS LARGE T ANTIGEN INTERACT WITH THE DISC AND PROTECT AGAINST FAS INDUCED APOPTOSIS

Rodier F.[1], Bertrand R.[1,2], and Mes-Masson, A-M.[1,2,*]

[1]Centre de recherche CHUM, Hôpital Notre Dame and Institut du cancer de Montréal, 1560 rue Sherbrooke est, Montréal, Québec, Canada H2L 4M1; [2]Département de médecine, Université de Montréal, Montréal, Québec, Canada
* Anne-Marie.Mes-Masson@UMontreal.CA

**INTRODUCTION**. Polyomavirus (Py), a member of the papovaviruses, causes tumors in rodents. Polyomavirus large T antigen (PyLT-Ag), a nucleophosphoprotein essential for regulating viral gene expression, modulates the cell cycle by binding to the Rb tumor suppressor gene product and immortalizes primary cells in culture. As part of their lytic cycle, viruses have developed strategies and appropriate viral gene products to either induce, delay, or totally block apoptosis (1,2). The receptor of FasL, CD95/Apo1/FasR, causes oligomerization of the FasR to provoke the formation of a death-inducing signaling complex (DISC) that transmits external apoptotic signals to the cytoplasm, mitochondria, and ultimately to the nuclei (3,4). Our laboratory has previously reported that a Sertoli cell line derived from transgenic mice expressing PyLT-Ag are resistant to treatment with FasR agonist antibodies (FasR(Ab)) (5). These results suggested that the inhibition of FasR(Ab)-induced apoptosis in these cells was attributable to PyLT-Ag expression. In this study, we investigated the effect of PyLT-Ag expression in murine cell lines sensitive to FasR(Ab) treatment.

**RESULTS**. Transfected TM4 (sertoli cell) clones showing strong PyLT-Ag expression (TM4-PGKLT) were used to test the effect of PyLT-Ag expression on apoptosis. Cells were treated with λ-INF for 16 h to induce FasR expression before FasR(Ab) was added. TM4-PGKLT cells were resistant to DNA fragmentation for at least 48 h compared to parental TM4 cells that underwent rapid DNA degradation after FasR(Ab) treatment. We analyzed the effect of PyLT-Ag expression on procaspase-8 and -3 activation in TM4 and TM4-PGKLT cells after FasR(Ab) treatment. The results clearly indicate that PyLT-Ag impedes the activation of procaspase-8 following FasR(Ab) stimulation (6). There was also a lack of early caspase-3 activity that seems to be a consequence of caspase-8 inactivation in PyLT-Ag expressing cells. To explain the lack of caspase-8 activation in FasR stimulated PyLT-Ag expressing cells, we investigated whether PyLT-Ag could interact with the DISC. In TM4-PGKLT cell extracts immunoprecipitated with antibodies against FADD the intensity of PyLT-Ag staining was greatly enhanced following the stimulation of FasR. As a control, PyLT-Ag was not observed following immunoprecipitations with goat pre-immune serum or with an anti-Hsp60 antibody (6).

**DISCUSSION/CONCLUSION**. We present evidence that PyLT-Ag protects cells from apoptosis induced by the stimulation of FasR. Analysis of caspase activation after FasR(Ab) stimulation in TM4 and TM4-PGKLT cells suggests that the protective effect of PyLT-Ag occurs primarily at or upstream of caspase-8, preventing its recruitment or activation to the FasR DISC. Our results suggest a function for PyLT-Ag in preventing caspase-8 activation in FasR(Ab)-treated cells because PyLT-Ag co-immunoprecipitates with FADD, and this interaction is strengthened following FasR activation and DISC formation. Since PyLT-Ag expression also prolongs the interval between caspase-3 activation and the occurrence of DNA fragmentation (6), it is possible that PyLT-Ag act downstream of caspase-3, delaying the activation of other caspases or nucleases that are involved in the structural disintegration of the nuclei and/or the DNA fragmentation processes. Thus, PyLT-Ag might act at different levels of the apoptotic cascade depending on its cellular localization. The ability of PyLT-Ag to protect cells from cell death receptor mediated apoptosis suggests that Py has possibly developed strategies to confer immune privileges that allow the host cell to escape immune responses. Further characterization of the mechanisms by which viral proteins like PyLT-Ag modulate apoptosis will help in understanding how tumor cells evade the immune system.

**ACKNOWLEDGEMENT**. A Medical Research Council of Canada grant to A.-M.M.-M funded this research.

**REFERENCES.**

1. Teodoro, J.G. and Branton, P.E. (1997) J. Virol. 71, 1739-1746
2. Young, L.S., Dawson, C.W., and Eliopoulos, A.G. (1997) Br. Med. Bull. 53, 509-521
3. Nagata, S. (1997) Cell 88, 355-365
4. Schmitt, E., Sane, A.T., and Bertrand, R. (1999) Drug Resist. Updates, 2, 21-29
5. Lebel, M., Bertrand, R., and Mes-Masson, A.M. (1996) Oncogene 12, 1127-1135
6. Rodier, F., Bertrand, R., Bossolasco, M., and Mes-Masson, A.M. (2000) Oncogene 19/54, 6261-6270
